# Cognition and behavior in adults with neurofibromatosis type 1

**DOI:** 10.3389/fneur.2024.1476472

**Published:** 2024-11-29

**Authors:** Anja Bos-Roubos, Hanneke van Leeuwen, Ellen Wingbermühle, Louisa van den Bosch, Lindsey Ossewaarde, Walter Taal, Laura de Graaff, Jos Egger

**Affiliations:** ^1^Centre of Excellence for Neuropsychiatry, Vincent van Gogh Institute for Psychiatry, Venray, Netherlands; ^2^Donders Institute for Brain, Cognition and Behaviour, Radboud University, Nijmegen, Netherlands; ^3^Center for Adults With Rare Genetic Syndromes, Erasmus University Medical Center, Rotterdam, Netherlands; ^4^Dialexis, Training Institute for Dialectical Behavior Therapy, Nijmegen, Netherlands; ^5^Department of Human Genetics, Radboud University Medical Centre, Nijmegen, Netherlands; ^6^Dutch Treatment Center Dialectical Behavior Therapy, Harderwijk, Netherlands; ^7^Eikenboom Psychosomatic Medicine, Altrecht Mental Health Institute, Zeist, Netherlands; ^8^Department of Neurology/Neuro-oncology, Erasmus MC Cancer Institute, University Medical Center, Rotterdam, Netherlands; ^9^ENCORE-Dutch Center of Reference for Neurodevelopmental Disorders, Rotterdam, Netherlands; ^10^Department of Internal Medicine, Erasmus University Medical Center, Rotterdam, Netherlands; ^11^Department of Internal Medicine, Radboud University Medical Center, Nijmegen, Netherlands; ^12^Stevig Specialized and Forensic Care for People With Intellectual Disabilities, Dichterbij, Oostrum, Netherlands

**Keywords:** neurofibromatosis type 1, NF1, congenital disorder, cognition, behavior, psychopathology, fatigue, contextual neuropsychology

## Abstract

**Background:**

Neurofibromatosis Type 1 (NF1) is a congenital neurocutaneous disorder. As NF1 is incurable and presents with a wide range of physical and mental symptoms, knowledge of neurocognitive and behavioral functioning can be an important aid in understanding their functional impact, and developing treatment options. To date, studies in children with NF1 have shown dysfunction in several domains, but much less is known about cognition and behavior in adults with NF1. The present study describes the neuropsychological phenotype of adults with NF1 based on comprehensive clinical examination of cognition and behavior across multiple functions.

**Methods:**

Participants were 62 adults with NF1 (mean age 38.2 years; *SD* 13.4). All underwent individual clinical neuropsychological assessment at the Center of Excellence for Neuropsychiatry as part of regular care. Scores on all individual measures were standardized into z-scores based on the corresponding normative group data. The proportions of mean z-scores in the NF1 study group were calculated according to cut-off points (±1 to ±1.5 SD; > ±1.5 SD) and compared to the expected proportions in the normal population distribution. Cognition and behavior was tested against population means constructed by bootstrapping.

**Results:**

Performance on the cognitive measures oral reading speed, visuospatial copying, visuospatial immediate recall, visual learning/imprinting, and visual memory immediate recall in the NF1 group were lower than normative means. The behavioral measures indicated higher levels of dysfunction, including psychopathology. The proportions of the NF1 study group with lower cognitive performance and higher levels of behavioral dysfunction were larger than in the normal population distributions. In addition, domain-level results revealed that intelligence, attention/speed, memory, and social cognition reflect cognitive dysfunction. Moreover, levels of emotion perception problems, experienced executive dysfunction, internalizing psychopathology (e.g., anxiety, depression), and severe fatigue were significantly higher compared to the simulated population sample. The mean level of emotion regulation (coping strategies) did not differ significantly from the population.

**Conclusion:**

Identified cognitive and behavioral dysfunction in multiple domains indicates high vulnerability in adults with NF1 and underscores the importance of individualized neuropsychological assessment and treatment. Further research on the relationships between cognition and behavior (including fatigue) in NF1 is warranted.

## 1 Introduction

Neurofibromatosis type 1 (NF1) is an autosomal dominant neurocutaneous disorder caused by variations in the *NF1* gene on chromosome 17q11.2 (1). This gene encodes Neurofibromin, which functions as a tumor suppressor by inhibiting activity of the *Ras/Mitogen-activated protein kinase (MAPK) pathway*. The estimated worldwide prevalence is 1:2,500–3,000 (2, 3). Between 40 and 75 percent of the variations arise *de novo* (4, 5).

The diagnosis of NF1 is often made clinically, in accordance with the international consensus on the diagnostic criteria (6, 7). Genetic testing may be needed if the clinical diagnosis is inconclusive, in the context of prenatal testing or in order to distinguish from other conditions.

NF1 is highly variable in its physical expression, and may manifest with pigmentary lesions (café-au-lait macules, skinfold freckling, and Lisch nodules), dermal neurofibromas, skeletal abnormalities (e.g., shortness of stature, scoliosis), peripheral nerve tumors (spinal neurofibromas, plexiform neurofibromas, and malignant peripheral nerve sheath tumors), and brain tumors (optic pathway gliomas and other pilocytic astrocytomas) (1). Specific structural and functional brain abnormalities include focal areas of signal intensity (FASI), increased brain volume, altered corpus callosum volume, cerebral asymmetries, and differences in white and gray matter volume (1, 8, 9). Other co-occurring physical symptoms may include hydrocephalus, seizures, headaches, cardiac abnormalities, cardiovascular disease, hypertension, vitamin D deficiency, and fatigue (10, 11). The progressive physical symptom profile of individuals with NF1 can change throughout their lives (10). As NF1 cannot be cured, medical treatment remains symptomatic. Due to the variety of signs and symptoms, some individuals with NF1 have little disease burden or are even unaware of their condition, while others suffer severely (11, 12).

People with NF1 may experience several neurocognitive and behavioral difficulties. Neurocognitive research to date has focused primarily on the pediatric NF1 population, with limited attention paid to adults. Nevertheless, a slightly lower than average full-scale Intelligence Quotient (IQ) has consistently been found across all ages, ranging from higher 80s to lower 90s (13–16).

In addition, deficits in visuospatial processing, visuospatial learning, attention, (nonverbal) working memory, planning, and other executive functioning have been widely reported in children (15–19). Lower visuospatial abilities and visuospatial memory have also been found in adults (13). Dysexecutive functioning (e.g., poor cognitive flexibility) has been reported in adults (13), but to a much lesser extent. One study has shown deficits in the executive control and no deficits in attentional function in adults (20). Lower academic achievement and poorer motor function have also been found in children (16, 19). In addition, deficits in language and speech development are common in children, and have been reported to some extent in adults (21, 22). In the pediatric population, deficits in social cognition and social functioning are commonly reported, in line with the increased prevalence of autism spectrum disorder (ASD) and attention-deficit hyperactivity disorder (ADHD) (23–25). In children, there is some evidence for deficits in facial emotion recognition, face perception (26, 27), and theory-of-mind (28). Studies of social-cognitive functioning in adults indicate potential deficits in emotion recognition and mentalizing (29, 30).

Despite the prevalence of brain abnormalities in NF1, brain imaging studies could not establish strong and consistent correlations between this morphology and cognitive functioning in NF1. Some functional magnetic resonance imaging (fMRI) research suggests possible associations between executive function deficits and dysfunction in the right inferior frontal areas and the middle frontal areas. In addition, visuospatial deficits may be related to dysfunction in the visual cortex, particularly in the magnocellular pathway involved in low spatial frequency and high temporal frequency processing (31). Furthermore, some connectivity studies have shown a reduction in anterior–posterior “long-range” connectivity and a deficit in the deactivation of the default mode network (DMN) during cognitive tasks (31).

Behavioral difficulties in NF1 include emotional distress and impaired social functioning. These challenges, along with high disease burden and reduced quality of life, affect individuals with NF1 of all ages, as well as their relatives (32–34). Significantly more symptoms of anxiety and depression have been reported in adults with NF1 compared to the population norm, even more than in patient groups with life-threatening diseases such as cancer (33, 35). Mood and anxiety problems in individuals with NF1 have been linked to the unpredictable course of NF1, the predisposition to develop malignancies, concerns about passing on NF1 to offspring, stigma, reduced social activity, deficits in prosocial behavior, lower self-esteem (34, 36, 58), and loneliness (22). In children and adolescents with NF1, concerns about sociability, school performance, psychological disorders, developmental (ASD, ADHD), emotional, and behavioral problems have been described; which also affect the well-being of their parents (32, 37, 38).

To summarize from the above, NF1 is not curable and is associated with a wide range of symptoms, both physical and mental. Previous studies in children and adolescents with NF1 have shown a variable neurocognitive phenotype with dysfunction in several domains, including attention, visuospatial processing, executive function, language, social cognition, developmental and behavioral problems. However, much less detailed information is known about cognition and behavior in adults with NF1 in one overview and study group.

To our knowledge, no detailed description of cognition and behavior in a large cohort of adults with NF1 exists to date. Knowledge of neurocognitive and behavioral functioning can be an important aid in analyzing symptom patterns, understanding their functional impact, and developing targeted treatment options. This study aims to describe the neuropsychological phenotype of adults with NF1 based on comprehensive, clinical examination of cognition and behavior across multiple functions.

Because behavior and psychopathology can be viewed as overlapping concepts, in this study we define behavior as the broader concept, referring to both functional and dysfunctional behavior. Psychopathology is defined as specific dysfunctional aspects of behavior in terms of mental and/or psychiatric illness.

In terms of expectations, based both on the literature as presented and on clinical practice, we hypothesize, first, that performance on individual cognitive tests in the NF1 group will be lower than the normative means of the instruments administered. Second, we expect higher levels of (experienced) behavioral problems compared to the normative means. Third, we expect the proportions in the NF1 group reflecting lower levels of cognitive performance and higher levels of behavioral problems compared to normal population distributions. Fourth, as in children, we expect the adult NF1 group to have cognitive function deficits and behavioral dysfunction across all cognitive and behavioral domains. In particular, we expect psychopathology (e.g., anxiety, depression) and high levels of fatigue.

## 2 Materials and methods

### 2.1 Participants

This study included 62 Dutch-speaking adults with NF1 with a mean age of 38.2 years (*SD =* 13.4; range 18–62). Table 1 shows sex and education of the participants. Data were collected between September 2017 and October 2022, as part of regular care. All participants underwent a comprehensive neuropsychological assessment at the Centre of Excellence for Neuropsychiatry, Vincent van Gogh Institute for Psychiatry, The Netherlands. Prior to their assessments, all participants or their legal representatives gave voluntary written informed consent for their data to be used anonymously for research purposes. No participants were excluded.

**Table 1:** Sex and education participants NF1.

	*n*	%
	62	
Sex		
Female	36	58.1
Male	26	41.9
Education^a^		
1	3	4.8
2	0	-
3	9	14.5
4	18	29.0
5	20	32.3
6	10	16.1
7	2	3.2

^a^ For level of education, we used a seven-point scale ranging from 1 (primary school not completed) to 7 (academic degree) according to the Dutch educational system (56). This scale is comparable to the International Standard Classification of Education (57).

In accordance with the diagnostic standards (6) the diagnosis of NF1 was clinically established in all participants; and, if medically indicated, also reconfirmed by DNA analysis. Of the participants 97% were referred to the Centre of Excellence for Neuropsychiatry after visiting the outpatient Center for Adults with Rare Genetic Syndromes, or the outpatient Clinic for Neurology/Neuro-oncology, both at the Erasmus University Medical Center, Rotterdam. The others (3%) were referred by their specialist at the Radboud University Medical Center, Nijmegen. All patients were referred because of symptoms of fatigue or psychosocial complaints without a (direct) physical explanation. None of the patients had known brain malformations according to the medical referral information.

The study was conducted according to the principles of the Declaration of Helsinki and was approved by the Vincent van Gogh Institutional Review Board (CWOP-EM/hl/2019.00.02/RvB/19.01818).

### 2.2 Materials

A comprehensive set of widely accepted standardized (neuro)psychological tests and questionnaires was administered to assess cognitive and behavioral abilities, distinguished by (11) domains, according to the classification of Lezak et al. (39): intelligence, attention/speed, executive function, verbal fluency, memory and learning, and social cognition, as well as levels of emotion perception problems, emotion regulation, subjective dysexecutive functioning, general psychopathology in terms of internalizing and externalizing psychopathology, and fatigue complaints. The Supplementary Figure S1 and Supplementary Table S1 provides an overview of the domains, the instruments administered, and corresponding references to normative data (appropriate for age, sex, and/or educational or intelligence level, if available).

### 2.3 Data collection

All of the cognitive and behavioral domains (including psychopathology) were assessed for each patient. The battery of tests used was variable on an individual level and tailored to the clinical questions of both the referring clinician and the patient. All tests were administered by trained psychologists, typically over a two-day period. There was no evidence of reduced mental effort during test administration for any of the participants. The following performance validity tests were administered: the Dutch test for short-term memory, i.e., Amsterdamse Korte Termijn Geheugen Test (AKTG; 62), the Test of Memory Malingering (TOMM; 63), or the Visual Association Test – Extended (VAT-E; 64). See also Supplementary Table S1, including the applied cut-off scores. Regarding symptom validity, we systematically analyzed all the embedded validity indicators of the Minnesota Multiphasic Personality Inventory (MMPI) following the procedure as described in Meyer and De Jonghe (64), and there was no indication for violation of the validity.

Informed consent forms were kept confidential and stored separately from test data in a locked cabinet at the Centre of Excellence for Neuropsychiatry. Transcripts linking a subject to a subject number were stored separately. Raw test data were obtained anonymously from a database at the Centre of Excellence for Neuropsychiatry. The data files at the Centre are accessible only to the researchers involved in the data collection and analysis of this study (AB-R, HVL, WO).

### 2.4 Statistical analysis

The data were analyzed at three levels: (1) at the level of outcome measures of the instruments administered; (2) at the level of proportions relative to the normal population distribution; (3) at the level of cognitive and behavioral domains to determine the presence or absence of dysfunction, using bootstrapping.

#### 2.4.1 Outcome measures

Participants’ scores on each individual test and questionnaire were calculated based on the normative data for each instrument (Dutch Advanced Neuropsychological Diagnostics Infrastructure (ANDI; 60) or data as presented in the respective test manuals). Raw scores on all tests and questionnaires were standardized to *z*-scores, using the normative group data (mean and standard deviation). Mean standardized scores for the NF1 study group were reported on each measure. For the Wechsler test battery, a two-tailed paired *t*-test of the means of the four indices was performed.

#### 2.4.2 Proportions

In addition, chosen cut-off points and corresponding classifications were 0 to ±1 SD (‘average’), ±1 to ±1.5 SD (‘below average’ or ‘above average’), and >±1.5 SD (‘low’ or ‘high’). Both wide and narrow cut-off point ranges were chosen to detect not only robust but also subtle differences in the NF1 study group compared to normative groups, and to avoid under- and over-estimation of their functioning. The percentages of the NF1 study group scores were then calculated according to the selected cut-off points. We calculated the proportions of the NF1 study group scores relative to the proportions of the theoretical normal distribution of the population.

#### 2.4.3 Domains

The convention in clinical neuropsychological practice is that a judgment about whether a function is impaired or not can only be made if it has been assessed with at least two tests (40). Therefore, in order to test our fourth hypothesis, we defined and compiled domains (41, 42). Twenty-seven Outcome measures from 13 cognitive tests administered were grouped into 6 different cognitive domains. Similarly, 22 outcome measures from 11 questionnaires administered were grouped into 5 different behavioral domains.

To test whether the NF1 study group differed from the general population, the observed *z*-scores of each participant were averaged per domain, resulting in mean domain *z*-scores per participant (*N =* 62), which were subsequently bootstrapped. Bootstrapping is an iterative replacement sampling procedure in which the observed scores are used to create proxy samples (“simulated population scores”). To construct the proxy sample (per domain), an observed mean domain score (from the NF1 study group subject) was randomly selected and copied. This process was repeated 1,000 times. The proxy samples have the same values but a slightly different composition than the NF1 study sample, as the observed scores are replaced in the study group pool to be selected again for simulation. A histogram of the means of a proxy sample approximates a standard distribution of means. The mean of this standard distribution is approximately equivalent to the mean of the observed study sample. However, the objective was not to determine whether the observed sample of participants with NF1 had the same mean as the “simulated population.” Rather, the aim was to determine whether the observed sample of participants with NF1 had the same mean as the general population of healthy controls. Since all scores of each instrument administered were previously compared to the corresponding normative controls and rescaled to *z*-scores (with a mean of 0 and a standard deviation of 1), we now assumed that the current domain scores also had a mean of 0. Therefore, the question was whether the observed mean was approximately equal to 0. This was tested by centering the standard distribution of the means of the created proxy samples around 0 and point estimating if our observed mean was still part of the 95% of proxy means around 0. If the observed mean falls outside this 95% interval, it can be assumed with sufficient certainty that the observed sample is not the same as a sample of healthy controls and that the domain score reflects either a strength or a weakness compared to healthy controls. The probability that the observed mean was part of the simulated population distribution with a mean of 0 was determined for each domain. Statistical analyses were performed in R Statistical Software [v.4.3.1; (43)].

## 3 Results

**Figure 1:**
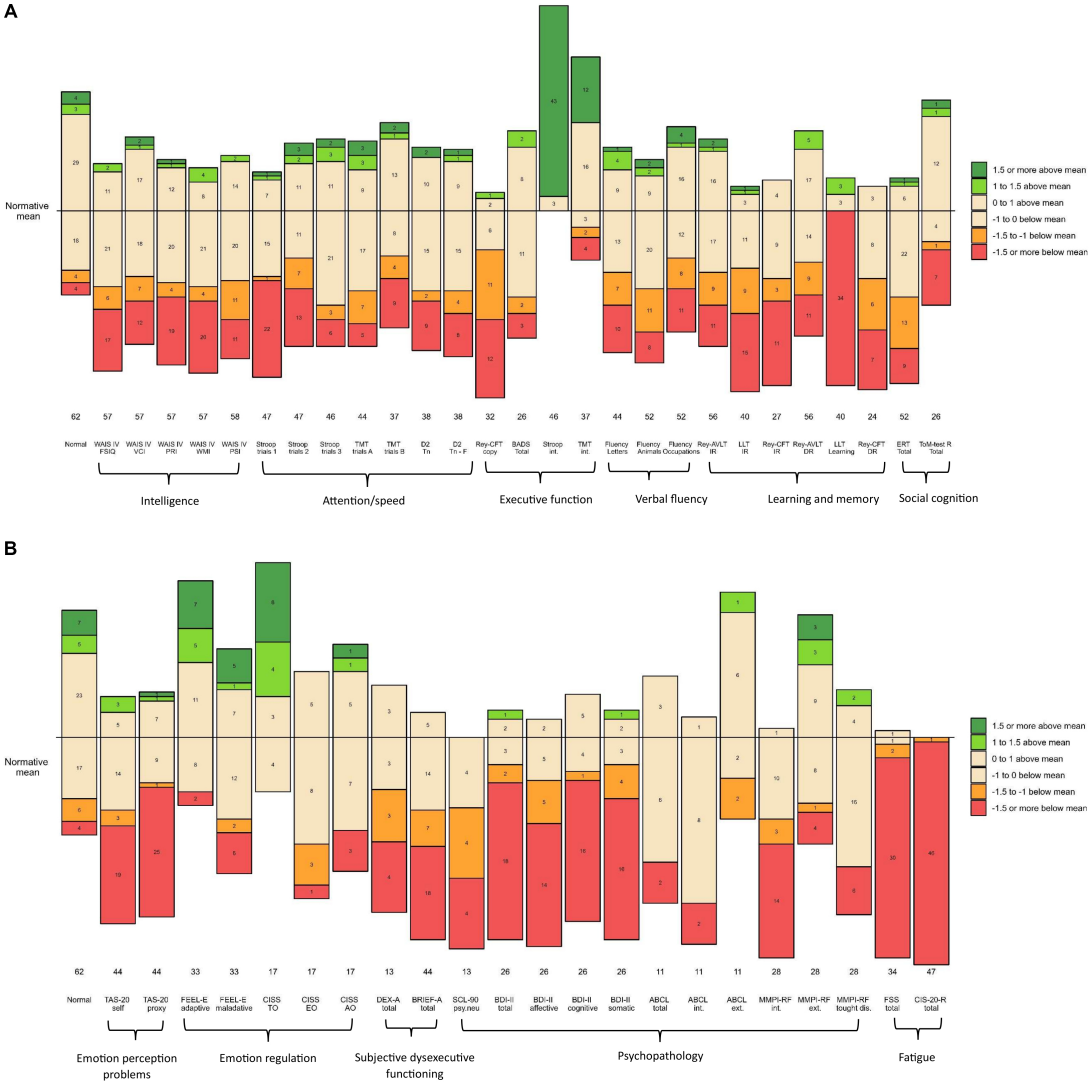
**(A)** Numbers of participants and *z*-scores on cognitive measurements compared to normative group. The horizontal line represents the normative mean based on the statistics described in the test manuals of the measures (normative controls). The numbers in the bars represent the number of participants in the corresponding range. The numbers below the bars reflect the size of the NFI sample on that measure. Performance is visualized based on the selected cut-off points (and classifications): 0 to ±1 (average); ±1 to ±1.5 SD (below or above average); ˃ ± 1.5 (=low or high) Orange and red colors indicate unfavorable compared to the normative mean. Green and dark green colors indicate favorable compared to normative mean. WAIS-IV-NL, Wechsler Adult Intelligence Scale-IV-NL; FSIQ, Full Scale Intelligence Quotient; VCI, Verbal Comprehension Index; PRI, Perceptual Reasoning Index; WMI, Working Memory Index; PSI, Processing Speed Index; TMT, Trail Making Test; D2, D2 Test of Attention; Tn, Total number; F, Faults; Rey CFT, Rey Complex Figure Test; BADS, Behavioral Assessment of the Dysexecutive Syndrome; Stroop, Stroop Color and Word Test; int., interference; ALVT, Auditory Verbal Learning Task; IR, Immediate Recall; DR, Delayed Recall; LLT, Location Learning Test; ERT, Emotion Recognition Task; ToM-R, Theory of Mind Test Revised. **(B)** Numbers of participants and *z*-scores on behavioral measurements compared to normative group. The horizontal line represents the normative mean based on the statistics described in the test manuals of the measures (normative controls). The numbers in the bars represent the number of participants in the corresponding range. The numbers below the bars reflect the size of the NF1 sample on that measure. Performance is visualized based on the selected cut-off points (and classifications): 0 to ±1 (average), ±1 to ±1.5 SD (below or above average); > ± 1.5 (=low or high). Orange and red colors indicate unfavorable compared to the normative mean. Green and dark green colors indicate favorable compared to normative mean. TAS-20, Toronto Alexithymia Scale-20; FEEL-E, Fragebogen zur Erhebung der Emotionsregulation bei Erwachsenen (Questionnaire for the survey of emotion regulation in adults); CISS, Coping Inventory for Stressful Situations; TC, Task oriented Coping; EC, Emotion oriented Coping; VC, Avoidance oriented coping; DEX, Dysexecutive Questionnaire; BRIEF-A, Behavior Rating Inventory of Executive Function-Adult Version; SCL-90, Symptom Checklist-90-R; psy.neu, psychoneurotic; BDI-II, Beck Depression Inventory-II; ABCL, Adult Behavior Checklist; int., internalizing; ext., externalizing; MMPI 2-RF, Minnesota Multiphasic Personality Inventory-2 Restructured Form; thought dis, thought disorders; FSS, Fatigue Severity Scale; CIS-20-R, Checklist Individual Strength-20-Revised.

Tables 2, 3 present all results in terms of the mean z-scores of the NF1 study group and the proportions of *z*-scores according to the cut-off points compared to the expected proportions based on normal population distributions. All scores are also shown graphically in Figure 1. The results are described in detail below, according to the tables layout.

**Table 2:** Results NF1 study group (Cognition).

	*N*	Mean *z* (SD)	Min. *z*	Max. *z*	Median *z*	% z ≤ −1.5	% z > −1.5 to −1.0	% z ≥ −1.0 to 0	% z ≥ 0 to 1.0	% z ≥ 1 to 1.5	% z ≥ 1.5
Classification of *z*-scores						Low	Below average	Average	Average	Above average	High
Expected % based on normal distribution in population						7%	9%	34%	34%	9%	7%
Measure											
Intelligence											
WAIS-IV FSIQ	57	−0.88 (1.11)	−3.47	1.27	−0.80	29.83	10.53	36.84	19.30	3.51	0.00
WAIS-IV VCI	57	−0.54 (1.06)	−2.67	1.93	−0.47	21.05	12.28	31.58	29.83	1.75	3.51
WAIS-IV PRI	57	−0.82 (1.15)	−3.27	1.93	−0.73	33.33	7.02	35.09	21.05	1.75	1.75
WAIS-IV WMI	57	−0.99 (1.27)	−3.47	1.47	−0.73	35.09	7.02	36.84	14.04	7.02	0.00
WAIS-IV PSI	58	−0.80 (1.17)	−3.67	1.13	−0.73	18.97	18.97	34.48	24.14	3.45	0.00
Attention/speed											
Stroop trials 1	47	−1.26 (1.52)	−4.44	1.5	−1.00	46.81	2.13	31.92	14.89	2.13	2.13
Stroop trials 2	47	−0.76 (1.52)	−3.96	2.16	−0.66	27.66	14.89	23.40	23.40	4.26	6.38
Stroop trials 3	46	−0.37 (1.05)	−2.64	1.96	−0.57	13.04	6.52	45.65	23.91	6.52	4.35
TMT A	44	−0.35 (1.19)	−3.77	2.29	−0.29	11.36	15.91	38.64	20.46	6.82	6.82
TMT B	37	−0.50 (1.17)	−2.84	1.75	−0.25	24.32	10.81	21.62	35.14	2.70	5.41
D2 Tn	38	−0.44 (4.05)	−2.00	2.00	−0.40	15.79	13.16	39.47	26.32	0.00	5.26
D2 Tn-F	38	−0.53 (4.12)	−2.00	1.60	−0.50	15.79	15.79	39.47	23.68	2.63	2.63
Executive function											
Rey-CFT Copy	32	−1.15 (0.78)	−2.06	1.32	−1.31	37.50	34.38	18.75	6.25	3.13	0.00
BADS battery total	26	−0.36 (0.95)	−2.29	1.22	−0.32	11.54	7.69	42.31	30.77	7.69	0.00
Stroop interference	46	3.23 (0.84)	0.07	3.87	3.38	0.00	0.00	0.00	6.52	0.00	93.48
TMT interference	37	0.41 (1.71)	−4.89	2.57	0.40	10.81	5.41	8.11	43.24	0.00	32.43
Verbal fluency											
Letters	44	−0.67 (1.36)	−3.92	1.68	−0.52	22.73	15.91	29.55	20.46	9.09	2.27
Animals	52	−0.64 (1.16)	−3.60	2.00	−0.66	15.39	21.15	38.46	17.31	3.85	3.85
Occupations	52	−0.45 (1.30)	−3.84	2.49	−0.34	21.15	15.39	23.08	30.77	1.92	7.69
Learning and memory											
*Immediate recall*											
Rey-ALVT – IR	56	−0.58 (1.13)	−2.83	2.75	−0.69	19.64	16.07	30.36	28.57	1.79	3.57
LLT – IR	40	−1.12 (1.07)	−3.04	1.92	−1.16	37.50	22.50	27.50	7.50	2.50	2.50
Rey-CFT – IR	27	−1.00 (0.93)	−2.78	0.86	−1.18	40.74	11.11	33.33	14.82	0.00	0.00
*Delayed recall*											
Rey-ALVT – DR	56	−0.59 (1.19)	−3.62	1.39	−0.43	19.64	16.07	25.00	30.36	8.93	0.00
LLT Learning	40	−2.08 (1.35)	−3.35	1.40	−2.48	85.00	0.00	0.00	7.50	7.50	0.00
Rey-CFT DR	24	−0.93 (0.80)	−2.58	0.80	−1.07	29.17	25.00	33.33	12.50	0.00	0.00
Social cognition											
ERT	52	−0.80 (0.95)	−2.71	1.62	−0.83	17.31	25.00	42.31	11.54	1.92	1.92
ToM-test-R	26	−0.35 (1.25)	−2.10	2.10	0.38	26.92	3.85	15.39	46.15	3.85	3.85

WAIS-IV-NL, Wechsler Adult Intelligence Scale-IV Dutch translation; FSIQ, Full Scale Intelligence Quotient; VCI, Verbal Comprehension Index; PRI, Perceptual Reasoning Index; WMI, Working Memory Index; PSI, Processing Speed Index; TMT, Trail Making Test; D2, D2 Test of Attention; Tn, Total number; F, Faults; Rey-CFT, Rey Complex Figure Test; BADS, Behavioural Assessment of the Dysexecutive Syndrome; Stroop, Stroop Color and Word Test; Rey-ALVT, Rey-Auditory Verbal Learning Test; IR, Immediate Recall; LLT, Location Learning Test; DR, Delayed Recall; ERT, Emotion Recognition Task; ToM-test-R, Theory of Mind test Revised.

**Table 3:** Results NF1 study group (Behavior).

	*N*	Mean *z* (SD)	Min. *z*	Max. *z*	Median *z*	% z ≤ −1.5	% z > −1.5 to −1.0	% z ≥ −1.0 to 0	% z ≥ 0 to 1.0	% z ≥ 1 to 1.5	% z ≥ 1.5
Classification of *z*-scores						Low	Below average	Average	Average	Above average	High
Expected % based on normal distribution in population						7%	9%	34%	34%	9%	7%
Measure											
Emotion perception problems											
TAS-20 self	44	1.13 (1.26)	−1.22	3.66	1.00	0.00	6.82	11.36	31.82	6.82	43.18
TAS-20 proxy	44	1.58 (1.67)	−1.64	4.92	1.81	2.27	2.27	15.91	20.46	2.27	56.82
Emotion regulation											
FEEL-E adaptive	33	−0.60 (3.70)	−3.00	3.00	−0.60	21.21	15.15	33.33	24.24	0.00	6.06
FEEL-A maladaptive	33	0.26 (3.56)	−3.00	3.00	0.30	15.15	3.03	21.21	36.36	6.06	18.18
CISS Task-oriented	17	−0.92 (0.81)	−2.00	0.46	−1.22	35.29	23.53	17.65	23.53	0.00	0.00
CISS Emotion-oriented	17	0.52 (0.72)	−0.58	2.40	0.36	0.00	0.00	29.41	47.06	17.65	5.88
CISS Avoidance- oriented	17	0.19 (1.23)	−2.61	2.29	0.18	5.88	5.88	29.41	41.18	0.00	17.65
Subjective dysexecutive functioning											
DEX total self	13	1.26 (1.75)	−0.97	5.13	1.08	0.00	0.00	23.08	23.08	23.08	30.77
BRIEF-A total self	44	1.37 (3.91)	−0.60	4.10	1.20	0.00	0.00	11.36	31.82	15.91	40.91
Psychopathology											
SCL-90 Psychoneuroticism	13	1.58 (1.57)	0.00	5.59	1.12	0.00	0.00	0.00	30.77	30.77	30.77
BDI-II total	26	2.98 (2.47)	−1.00	7.97	2.29	0.00	3.85	7.69	11.54	7.69	69.23
BDI-II affective	26	2.45 (2.46)	−0.60	9.47	2.09	0.00	0.00	7.69	19.23	19.23	53.85
BDI-II cognitive	26	2.63 (2.64)	−0.60	8.06	2.22	0.00	0.00	19.23	15.39	3.85	61.54
BDI-II somatic	26	2.52 (2.07)	−1.12	6.53	2.14	0.00	3.85	7.69	11.54	15.39	61.54
ABCL total self-report	11	0.54 (4.02)	−0.60	2.70	0.30	0.00	0.00	27.27	54.55	0.00	18.18
ABCL internalizing self-report	11	0.96 (3.95)	−0.20	3.00	0.80	0.00	0.00	9.09	72.73	0.00	18.18
ABCL externalizing self-report	11	−0.07 (4.24)	−1.20	1.10	−0.30	0.00	9.09	54.55	18.18	18.18	0.00
MMPI-2-RF internalizing (rEID)	29	1.63 (3.81)	−0.90	3.90	1.50	0.00	0.00	3.57	35.71	17.86	42.86
MMPI-2-RF externalizing (rBXD)	29	0.09 (3.92)	−1.60	2.40	−0.10	10.71	10.71	32.14	28.57	3.57	14.29
MMPI-2-RF thought disorders (rTHD)	29	0.86 (3.57)	−1.20	4.80	0.70	0.00	7.14	14.29	57.14	0.00	21.43
Fatigue											
FSS total	34	3.99 (1.81)	−0.14	6.71	4.49	0.00	0.00	2.94	2.94	5.88	88.24
CIS-20-R total	47	2.63 (0.81)	1.14	4.52	2.40	0.00	0.00	0.00	0.00	2.13	97.87

TAS-20, Toronto Alexithymia Scale-20; FEEL-E, Fragebogen zur Erhebung der Emotionsregulation bei Erwachsenen (Questionnaire for the survey of emotion regulation in adults); CISS, Coping Inventory for Stressful Situations; TO, Task Oriented; EO, Emotion Oriented; AO, Avoidance Oriented; DEX, Dysexecutive Questionnaire; BRIEF-A, Behaviour Rating Inventory of Executive Function- Adult Version; SCL-90, Symptom Checklist-90-R; BDI-II, Beck Depression Inventory-II; ABCL, Adult Behaviour Checklist; MMPI-2-RF, Minnesota Multiphasic Personality Inventory-2 Restructured Form; FSS, Fatigue Severity Scale; CIS-20-R, Checklist Individual Strength-20-Revised.

As to intelligence, the mean Full-Scale Intelligence Quotient (FSIQ) on the Wechsler Adult Intelligence Scale IV-NL (WAIS-IV-NL) was 86.82 (*SD* = 16.67), ranging from 48 to 119 (median FSIQ 88.00). The level of the mean observed FSIQ was approximately one standard deviation lower than the mean FSIQ of the normative population (*M =* 100; *SD =* 15). The means of the four indices of the WAIS-IV-NL of the NF1 group were: Verbal Comprehension Index (VBI) 91.91 (*SD =* 15.93), Perceptual Reasoning Index (PRI) 87.74 (*SD* = 17.23), Working Memory Index (WMI) 85.19 (*SD =* 19.00), and Processing Speed Index (PSI) 87.95 (*SD =* 17.55). The mean VBI was significantly higher than the mean WMI (*t =* 2.42, *df =* 56, *p =* 0.019), and higher than the mean PRI (*t =* 3.67, *df =* 56, *p <* 0.001). In addition, all mean WAIS-IV-NL z-scores of the NF1 study population were overrepresented at the lower end of the normal distribution. They were also underrepresented at the upper end of the normal distribution.

With respect to attention/speed, 6 of the 7 measures composing this domain, had mean z-scores below zero, but remained within the average range (−1.0 to 0). The mean z-score of one measure, related to oral reading speed (Stroop test trial 1), was below average. In addition, almost half of the z-scores on this measure in the entire NF1 sample (46.8%) were low (*z ≤* −1.5). Moreover, the observed attention/speed scores in the NF1 sample were overrepresented at the lower end of the normal distribution compared to the normative group. There was also an underrepresentation of observed scores at the higher end of the normal distribution.

In the executive functioning domain, the mean z-scores of 2 (Stroop interference, Trail-Making-Test interference) of the 4 measures were above zero. These two measures relate to interference sensitivity. The proportion of these observed z-scores was over-represented at the upper end of the normal distribution in the normative group. This means that the performance of the study group was remarkably good at this point. The results of the other 2 (Rey-Complex Figure Test Copy; Rey-CFT Copy, and Behavioral Assessment of the Dysexecutive Syndrome; BADS) of the 4 executive function measures were negative. These two measures relate to several cognitive functions, such as planning and organization, fine motor coordination, and visuospatial perception. In particular, the mean z-score of the test condition related to fine-motor coordination and visuospatial perception (Rey-CFT Copy) reflected below average performance. Almost three-quarters (71.9%) of the z-scores of the entire NF1 sample on this test condition were in the below average and low range (*z <* −1). In addition, the observed scores on the Rey-CFT Copy were overrepresented at the lower end of the normal distribution relative to the normative group. Underrepresentation of scores at the upper end of the normal distribution was also observed for both the Rey-CFT Copy and the BADS.

Regarding verbal fluency, the mean z-scores of the 3 function measures in this domain were all below zero, but still in the average range (between −1 and 0). However, the observed scores were again overrepresented at the lower end of the normal distribution compared to the normative group. Also, the observed scores were predominantly underrepresented at the upper end of the normal distribution.

Within the learning and memory domain, the mean *z*-scores of the 6 measures were all below zero, ranging from −0.58 to −2.08. In particular, the mean z-score of the visual learning test (Location Learning Test, Learning; LLT Learning) was low (*z =* −2.08). On this visual learning test, 85% of the total NF1 sample had a z-score below −1.5, indicating low performance in the majority of the NF1 sample. Regarding the (3) memory measures in the immediate recall condition (requiring preceding learning and imprinting), the mean z-scores of the two visual conditions (LLT Immediate Recall, CFT-Rey Immediate Recall) were below average. In contrast, the mean z-score of the auditory condition (Auditory Verbal Learning Test, Immediate Recall; AVLT Immediate Recall) was in the average range, although below zero (*z =* −0.58). The observed z-scores on all (3) Immediate Recall memory tests were overrepresented in both the below-average and the low ranges. More specifically, a total of 60% of the NF1 sample had z-scores lower than −1 on the LLT Immediate Recall. A total of 51.8% of the NF1 sample had z-scores lower than −1 on the CFT-Rey Immediate Recall. The immediate recall performance of the NF1 sample was also underrepresented at the upper end of the normal distribution, particularly on the two visual immediate recall tests, both in the above average and in the high range. With respect to the Delayed Recall condition of memory (indicating the ability to retain and recall stored information) of which the performance is related to the amount of imprinted information, mean z-scores on these two tests were both below zero, but still within the average range. On the visual delayed recall condition (CFT-Rey Delayed Recall) (mean *z =* −0.93), a total of 54.17% of the NF1 sample had z-scores lower than −1. On the auditory delayed recall condition (AVLT Delayed Recall), a total of 35.71% of the NF1 sample had z-scores lower than −1. In both the visual and auditory delayed recall conditions, the performance of the NF1 sample was overrepresented at the lower end of the normal distribution. In addition, the delayed recall performance of the NF1 sample was also underrepresented at the upper end of the normal distribution.

In terms of social cognition, the mean *z*-scores of the two measures were both below zero, but remained in the average range. However, the observed scores in the NF1 sample on both measures were overrepresented at the lower end. On the Emotion Recognition Task (ERT), 42.31% of the NF1 sample scored below average and low, compared to 12% of the normative group. Similarly, on the Theory of Mind Test, 30.77% of the NF1 sample scored low and 3.85% scored below average; most of them (26.92%) low, compared to 7% of the normative group. Again, the observed scores of the NF1 sample were underrepresented at the upper end of the normal distribution.

Regarding the perception of one’s own emotions, the mean level of experienced problems (as inventoried by the Toronto Alexithymia Scale-20 self; TAS-20 self) was above average (*z =* 1.13). Half of the entire NF1 sample (50%) had above average and high levels of problems compared to 16% in the normative group. Underrepresentation was also observed at the lower end of the normal distribution. With respect to the regulation of emotions (the ability to deal with emotions as inventoried by the *Fragebogen zur Erhebung der Emotionsregulation bei Erwachsenen;* FEEL-E and the Coping Inventory for Stressful Situations; CISS), the two mean z-scores for adequate coping ranged from −1 to 0, while the mean z-scores for inadequate coping ranged from 0 to 1. Although all mean z-scores of emotion regulation in the NF1 sample were in the average ranges, inadequate emotion regulation was overrepresented at above average and at high levels and adequate emotion regulation was underrepresented at below average and at low levels in the NF1 group compared to the normative group.

In terms of subjective dysexecutive functioning, the mean z-scores indicated that the NF1 group experienced executive problems at above average to high levels. More than half of the NF1 sample reported dysexecutive functioning at above average to high levels [53.85% on the questionnaire Dysexecutive functioning (DEX) and 56.82% on the Behavior Rating Inventory of Executive Function-Adult Version (BRIEF-A), compared to 16% of the normative group], with most at the high level.

Regarding psychopathology, the mean z-scores on the measures of internalizing pathology Complaints List (SCL-90), Beck Depression Inventory-Second Edition (BDI-II Total), MMPI-2-RF Emotional-Internalizing Dysfunction (MMPI-2-RF rEID) were all high (*z* > 1.5). Specifically, on the SCL-90 30.77% of the observed Z-scores in the total NF1 sample were above 1.5; on the BDI-II Total 69.23%; and on the MMPI-2-RF Internalizing behaviors 42.86% of (all compared to 7% in the normative group). Mean z-scores for externalizing pathology (MMPI-2-RF rBXD) and thought disorders (MMPI-2-RF rTHD) were above zero, but at average levels. However, 14.29% of the entire NF1 sample reported symptoms of externalizing pathology at a high level, and 21.43% of them symptoms of thought disorders at a high level, compared to 7% of the normative group.

With respect to fatigue, the mean z-scores of the two measures (Fatigue Severity Scale; FSS and Checklist Individual Strength; CIS-20-R) ranged from 2.6 to 4. Almost the entire NF1 sample (88.2% and 97.9%, respectively) reported fatigue at high levels.

**Figure 2:**
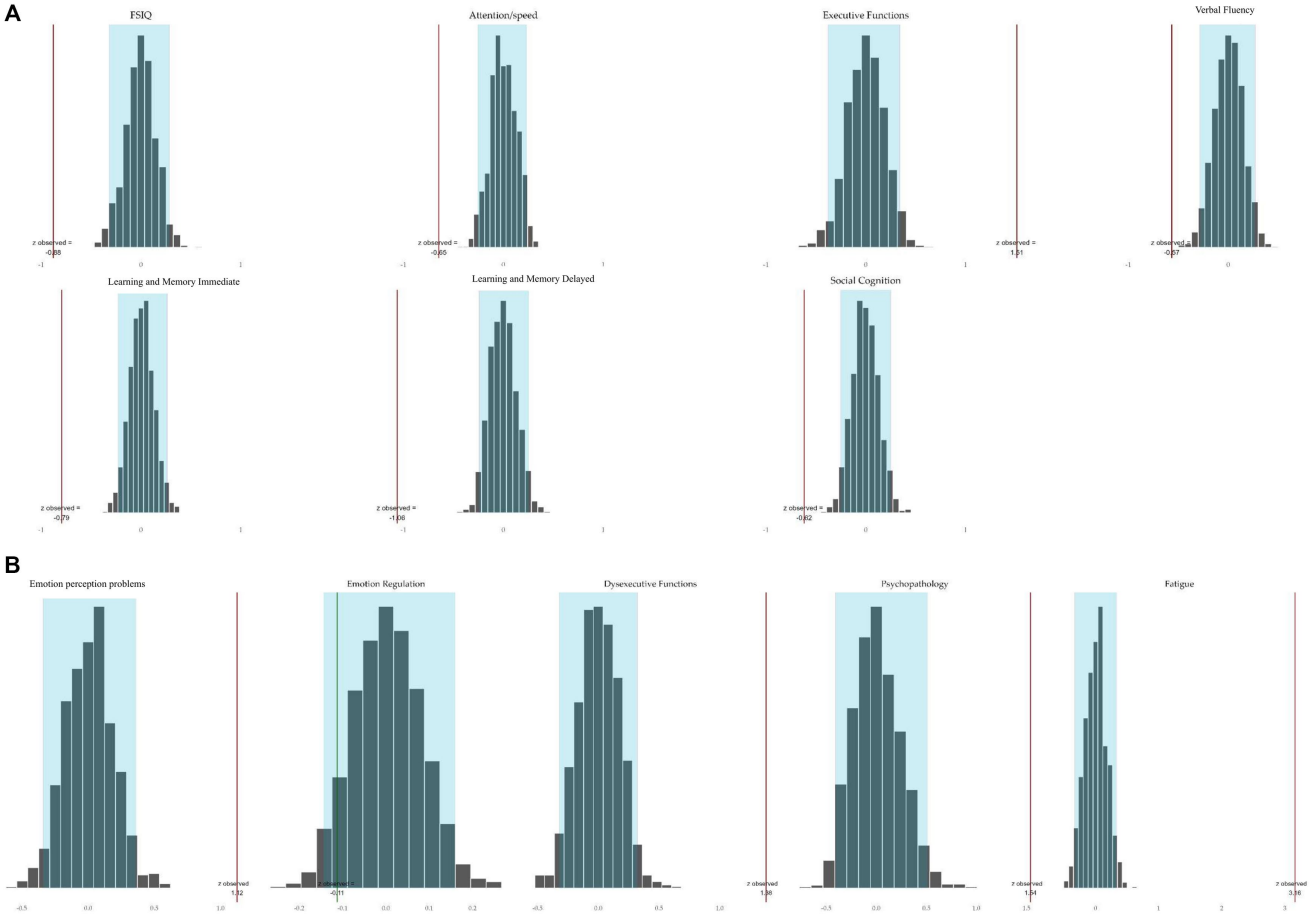
Bootstrapped simulated population domain distributions and mean observed domain *z*-scores of the NF1 study sample tested on 95% confidence level. (A) Cognition. The red bar to the left of the distribution indicates that the observed mean of the NFI group was significantly lower than 0. A red bar to the right of the distribution indicates that the mean performance of the NF1 group was significantly higher than 0. (B) Behavior. The red bar to the right of a distribution indicates that the observed mean of the NFI group was significantly higher than 0. The green bar in the distribution indicates that the observed mean of the NF1 group was not significantly different from 0.

For cognition and behavior tested at the domain-level using bootstrapping, the observed mean z-scores of the NF1 study sample per domain did not fall all within the values of the population distribution per domain. As shown in Figure 2, the blue boxes in this normal distributions represent 95% of the population scores with a mean of 0. The bars reflect the observed NF1 study group mean z-score per domain. With at least 95% confidence, the probability that the NF1 sample means of all the other domains were part of the population distribution with a mean around 0 is less than <5% (*p <* 0.05). The mean z-scores of the NF1 study group differed significantly from the population on all cognitive and behavioral domains, except for the domain emotion regulation. Specifically, the mean domain performances on intelligence (FSIQ), attention/speed, verbal fluency, learning and memory (immediate and delayed), and social cognition were lower. Consequently, these domains may be considered to reflect cognitive weaknesses in the NF1 study group. In addition, the mean performance was higher in the executive function domain, driven by lower interference sensitivity, which may therefore be considered a relative strength. The mean observed z-scores of the domains emotion perception problems, subjective dysexecutive functioning, psychopathology, and fatigue were higher, indicating the presence of behavioral weaknesses in the NF1 sample. In contrast, the domain mean emotion regulation did not differ from the population mean.

## 4 Discussion

This study describes the neuropsychological phenotype of adults with NF1.

In terms of cognitive functioning, performances of the NF1 group were lower than the normative means on the following measures: oral reading speed, visuospatial perception, visual learning, and visual memory (immediate recall). Behavioral functioning measures revealed higher levels of emotion perception problems, subjective dysexecutive functioning, internalizing psychopathology (anxiety, depression), and fatigue in the NF1 group. These findings are consistent with our first and second hypotheses.

In addition, the proportions of the NF1 group with lower levels of cognitive functioning and higher levels of behavioral problems were larger than in the normal population distributions. This is consistent with our third hypothesis. These larger proportions were true for almost all cognitive outcome measures (except the 2 measures of interference sensitivity), and for all behavioral outcome measures.

Fourth, the NF1 group appeared to have lower levels of intelligence, and cognitive dysfunctions in attention/speed, verbal fluency, learning and memory, and social cognition. Executive functioning (in terms of planning, organization, and inhibitory control) was not impaired in our sample, contrary to our expectations. In terms of behavior, the mean levels of emotion perception problems, experienced dysexecutive functioning, psychopathology, and fatigue were significantly higher in the NF1 group than in the general population. In contrast, the mean level of emotion regulation in the NF1 group did not differ from the population.

With respect to the domain of executive functioning, low interference sensitivity as we found in the adult NF1 group is consistent with the previous finding of low interference sensitivity in children with NF1 (17). However, interference sensitivity was measured by a task that depends in part on information processing speed. Therefore, the finding that the NF1 group was not affected by interference sensitivity, may be, at least in part, explained by their slower information processing speed (38% of the NF1 group had PSI scores below the cut-off point of 1 SD). As a consequence, low sensitivity to interference in both children and adults may seem to reflect a strength in executive functioning, but is primarily driven by slowness, which drives up the domain score. Therefore, the effect of interference sensitivity is difficult to be measured properly.

Memory function measures show that delayed recall performance was less impaired than immediate recall performance. Again referring to the slower speed, this finding may reflect that people in the NF1 study group need more time to process and consolidate information in both learning and memory conditions. Moreover, both learning and memory in the NF1 sample were likely affected by deficiencies in visual information processing, considering the fact that results on primary measures of visuospatial information processing were low. Impairments in visuospatial processing and visuospatial memory have been consistently found in both children and adults with NF1 (13). Congruent to this, performances on visual memory tasks were, in our sample, worse than those on auditory/verbal learning and memory tasks. This finding is also consistent with previous studies showing nonverbal learning problems in adults (44) and (nonverbal) (working) memory problems in children (19).

Regarding the verbal fluency deficits in our study group, the literature is conflicting. Reduced verbal fluency has been found in adults with NF1 (13, 45, 46), while the opposite has also been reported (47). Findings on verbal fluency in children with NF1 are also inconsistent. Speech/expressive language problems have previously been reported in (very young) children with NF1 (48, 49), while other studies have found no significant differences in children with NF1 compared to a comparison group (14, 65). It should be noted that the results may vary to some extent due to the different definitions of “verbal fluency,” the different test instruments used, and/or the cut-off points chosen.

Impairments in social cognition as demonstrated in our study group are consistent with previous findings in children, who typically show difficulty with facial emotion recognition and theory of mind (26–28).

In terms of behavior, the fact that emotion regulation in our NF1 group did not differ significantly from the population mean is contrary to our expectations, as emotional problems have been found in previous studies with children with NF1 (32, 37, 38). This finding is also inconsistent with our clinical observations of difficulty with emotion regulation in individuals with NF1. A closer look at the different measures within the emotion regulation domain revealed that the proportions of maladaptive coping were higher and the proportions of adaptive coping in the NF1 group were lower than those in the normative group. In particular, the study sample showed little active and task-oriented coping, with a concomitant tendency to experience emotions without being able to differentiate them properly. Thus, although significance levels were not reached for emotion regulation at the domain level, coping of these NF1 patients can be characterized as predominantly passive in nature, possibly contributing to the high levels of psychopathology that were found in this study sample.

While the recognition and expression of one’s own emotions have yet to be studied in adults with NF1, alexithymia has been demonstrated repeatedly in other RASopathies, such as Noonan syndrome (50, 51). Consequently, our results for the NF1 group regarding this trait are rather novel, yet not necessarily surprising.

Finally, levels of internalizing psychopathology (e.g., anxiety, depression) and fatigue appear to be remarkably high in the study sample, even when considering that medically unexplained fatigue was the reason for referral. Anxiety and depression in adults with NF1 have been consistently reported in the literature (33, 35). High levels of experienced fatigue are consistent with the literature in children and adolescents (61). High levels of fatigue in adults with NF1 were not related to their somatic conditions, suggesting that fatigue may be induced and/or maintained by cognitive and psychological rather than physical conditions (11).

Overall, cognitive and behavioral performance of the NF1 study group on many measures differed from the normative population. The proportions of the NF1 study group with lower than expected cognitive test scores and higher levels of behavioral problems were higher than in the normal population distribution. In addition, domain testing revealed impaired intelligence, attention/speed, memory, and social cognition. In addition, the levels of emotion perception problems, experienced executive dysfunction, internalizing psychopathology (e.g., anxiety, depression), and fatigue can be considered as significantly high compared to the expected/simulated population group mean. There were no behavioral strengths according to our measurements.

A notable limitation of the study is that all participants in the study were referred to the outpatient clinic of the Centre of Excellence for Neuropsychiatry by their medical specialist because of fatigue or other psychosocial symptoms that could not be sufficiently explained somatically. This may have entailed a selection bias. It is possible that the average level of cognitive performance may be lower and the average level of behavioral dysfunction may be higher in this sample than in individuals with NF1 who experience lower levels of fatigue or psychosocial symptoms. Related to this, it is also relevant to note that only patients with NF1 with somatic disorders in the course of their genetic condition were referred, and there was no control group of patients without such somatic burdens or complaints. This weakness may limit the generalizability of the results to the NF1 population in general. However, is it is challenging to avoid a certain degree of selection bias in patient studies in the field of rare genetic disorders. Moreover, several characteristics of the NF1 sample (such as the FSIQ as established) are consistent with those of other studies and clinical observations. In addition, the study group was large for its kind, and demographic variables like gender and education level were equally distributed in this NF1 sample.

As a strength of this study can be mentioned that the neuropsychological profile of this NF1 group was comprehensively and extensively mapped, and appropriate analytical methods were deliberately selected. The selected cut-off points revealed both subtle (1 to 1.5 SD) and robust (more than 1.5 SD) deficits. Phenotyping the NF1 study sample at different levels of analysis revealed that the proportion of unfavorable performance is overrepresented and the proportion of favorable results is underrepresented. This could have been easily overlooked if only the mean scores of the instruments administered had been inspected. The distributions in the NF1 group as described indicate multiple cognitive and behavioral vulnerabilities. Furthermore, analysis at the function domain levels (conventionally at least 2 neuropsychological tests) also revealed dysfunction across multiple domains.

For future studies, it is important to include comparison groups in the neuropsychological phenotyping of NF1. Given that both genetic variability and symptom expression are highly variable in NF1, adults with NF1 without fatigue as well as IQ-matched adults with and without fatigue could be valuable control subjects. As part of the wide range of behavioral problems, our study group had very high levels of fatigue. To further address this issue, a future study could analyze the potential associations and interactions between fatigue and cognition and behavior, and determine whether the fatigue symptoms can be statistically explained by cognitive and behavioral dysfunction. Cognitive dysfunction and behavioral difficulties, such as inadequate coping, are likely to impair an individual’s ability to engage effectively in occupational and daily activities and to meet (self-imposed and societal) expectations. In addition, deficits in social cognitive functioning, such as impaired perception and regulation of emotions and impaired mentalizing skills, have been linked to internalizing psychopathology, including mood disorders (29, 30, 52). Symptoms of fatigue (both physical and mental) in NF1 may be bidirectionally related to cognitive impairment and behavioral dysfunction (11, 53). Addressing the interconnectedness of NF1 issues requires a comprehensive (research and clinical) approach that analyses the interplay between cognitive function and behavior, including coping mechanisms, and mental well-being (54).

Our findings underscore the importance of individualized clinical neuropsychological assessment in individuals with NF1 who present with symptoms that cannot be adequately explained medically. It is also prudent to consider that cognitive deficits are not always immediately apparent or quantifiable in everyday life and may therefore go unnoticed. Due to the concomitant absence of externalizing psychopathology, cognitive and behavioral dysfunction of individuals with NF1 may not attract immediate clinical attention. However, in the absence of sufficient adaptive coping, the risk of psychopathology, and high levels of fatigue, clinicians should be alert to even (subtle) cognitive and behavioral symptoms (e.g., a history of learning problems, lower verbal participation in oral conversation, withdrawal or delayed follow-up of treatment or appointments, and perceiving own NF1 symptoms or burden as predominantly physical with less attention for the mental aspects) and refer for clinical neuropsychological assessment. Based on individual assessment, personalized treatment options can be provided. For example, evidence-based dialectical behavior therapy (DBT) tailored to adults with NF1 in the Netherlands (55), tailored psychoeducation (for individual patients and their proxies), cognitive compensation strategies, and evidence-based treatment of anxiety and depression can be offered, along with interventions aimed at optimizing and adapting the environment to the capabilities of the individual with NF1.

In summary, this study comprehensively inventoried and tested cognition and behavior in a large group of adults with NF1 across a broad age range, in accordance with the gold standard for individualized clinical neuropsychological assessment. In line with brain imaging research, that has not yet shown robust correlations between specific brain morphologies and dysfunction in NF1, we found dysfunction in adults across multiple domains.

## Data Availability

The data that support the findings of this study are available from the corresponding authors (AB-R, HL) upon reasonable request.
