# Supplementary material for: Cognition and behavior in adults with neurofibromatosis type 1
Source: Front Neurol. 2024 Nov 29;15:1476472. doi: 10.3389/fneur.2024.1476472 (PMC11638057; doi:10.3389/fneur.2024.1476472)
Supplement: Supplementary file 1 [file Data_Sheet_1.pdf]

Supplementary Figure 1. Overview of cognitive and behavioral domains and instruments administered

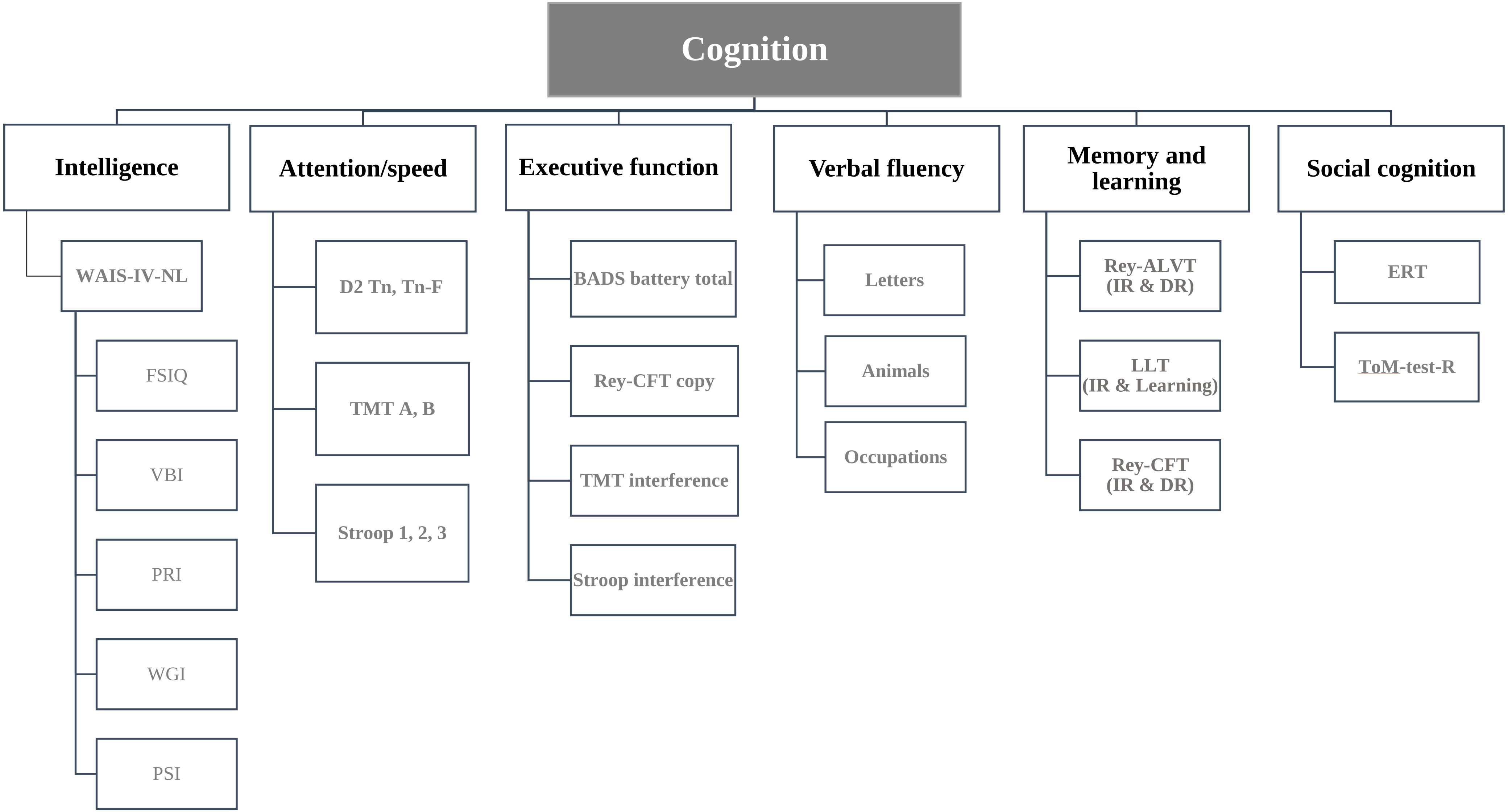

October 22, 2024

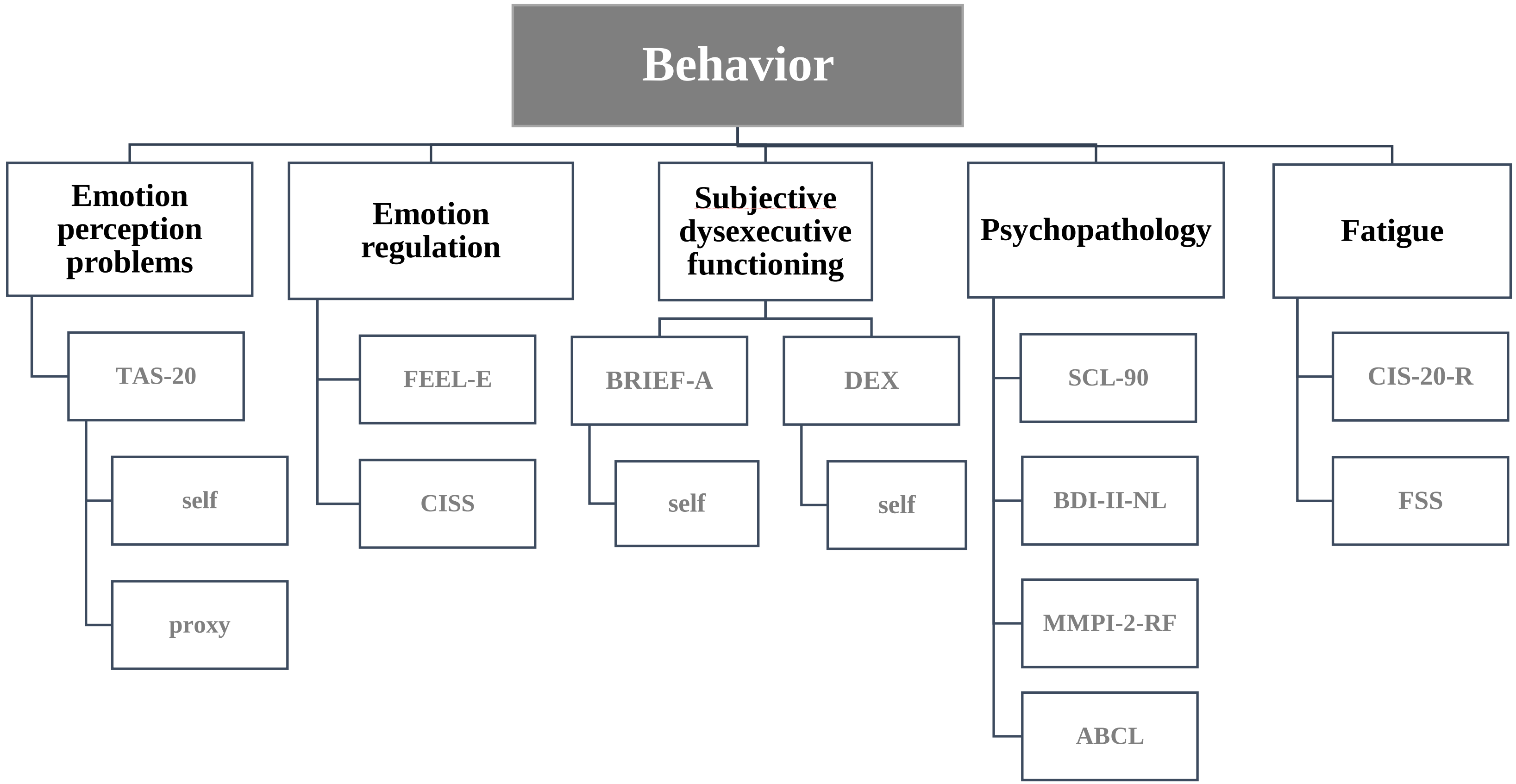

Supplementary Figure 1.
Abbreviations: WAIS-IV-NL: Wechsler Adult Intelligence Scale-IV-NL; FSIQ: full scale intelligence quotient; VBI: verbal comprehension index; PRI: perceptual reasoning index; WGI: working memory index; PSI: processing speed index; D2: D2 Test of Attention; TMT: Trail Making Test; Stroop: Stroop Color and Word Test trials 1,2, and 3; Rey-CFT; Rey-Osterrieth Complex Figure Test; BADS: Behavioural Assessment of the Dysexecutive Syndrome; Rey-ALVT: Rey Auditory Verbal Learning Test; IR: Immediate Recall; DR: Delayed Recall; LLT: Location Learning Test; ERT: Emotion Recognition Task; ToM-test-R: Theory-of-Mind test Revised. TAS-20: Toronto Alexithymia Scale-20; FEEL-E: Fragebogen zur Erhebung der Emotionsregulation bei Erwachsenen (Questionnaire for the survey of emotion regulation in adults); CISS: Coping Inventory for Stressful Situations; BRIEF-A: Behaviour Rating Inventory of Executive Function- Adult Version; DEX: Dysexecutive Questionnaire; SCL-90: Symptom Checklist-90-R; BDI-II: Beck Depression Inventory-II; MMPI 2-RF: Minnesota Multiphasic Personality Inventory-2 Restructured Form; ABCL: Adult Behaviour Checklist; FSS: Fatigue Severity Scale; CIS-20-R: Checklist Individual Strength-20-Revised.

Supplementary Table 1. Overview of neuropsychological instruments, abbreviations, and references

**Neuropsychological instrument**	**Abbreviation**	**Reference**
Amsterdamse Korte Termijn Geheugen Test *(Dutch short-term memory test)*	AKTG	Schmand, B., De Sterke, S., & Lindeboom, J. (1998). *AKTG : Amsterdamse korte termijn geheugen test*. Lisse: Swets Test Publishers.<br>Cut-off point: ≤84
Test of Memory Malingering	TOMM	Tombaugh, T. N. (1996). *Test of Memory Malingering (TOMM)*. New York: MHS.<br>Tombaugh, T. N. (2013). *Test of memory malingering TOMM; user's manual*. New York: MHS.<br>Cut-off points: trial 1 < 18; trial 2 < 45
Visual Association Test – Extended	VAT-E	Meyer, S. R. A., & De Jonghe, J. F. M. (2019). *Visuele associatietest – Extended*: Handleiding. Amsterdam: Hogrefe Uitgevers BV.<br>Meyer, S. R. A., De Jonghe, J. F. M., Schmand, B., & Ponds, R. W. H. M. (2017). The Visual Association Test – Extended: A cross-sectional study of the performance validity measures. *The Clinical Neuropsychologist*, *31*(4), 798-813. https://doi.org/10.1080/13854046.2017.1280181<br>Cut-off points: ≤ 21 immediate recognition; ≤ 20 delayed recognition; ≤ 21 consistency; ≥ 7 free recall combined with ≤ 9 multiple choice
Wechsler Adult Intelligence Scale IV-NL	WAIS-IV-NL	Wechsler, D. (2012). *Wechsler Adult Intelligence Scale – Fourth Edition, Nederlandstalige Bewerking* (WAIS-IV-NL): Technische handleiding *(WAIS-IV Dutch version: Technical manual)*. Amsterdam: Pearson.
Rey Auditory Verbal Learning Test (*Dutch translation:* 15-Woorden Test)	AVLT	Rey, A. (1964). *Rey auditory verbal learning test (RAVLT)*. L'Examen clinique en psychologie *[Clinical tests in psychology]*. Paris: Presses Universitaires de France (PUF).<br>Bouma, A., Mulder, J., Lindeboom, J. & Schmand, B. (2012). Handboek Neuropsychologische Diagnostiek (tweede herziene druk) *[Handbook neuropsychological assessment]*. Amsterdam: Pearson.
Rey-Osterrieth Complex Figure Test	Rey CFT	Lezak, M. D., Howieson, D. B., Bigler, E. D., & Tranel, D. (2012). *Neuropsychological Assessment (5th ed)*. New York: Oxford University Press.<br>Bouma, A., Mulder, J., Lindeboom, J., & Schmand, B. (2012). *Handboek Neuropsychologische Diagnostiek (tweede herziene druk)*. Amsterdam: Pearson Assessment and Information B.V.
Location Learning Test	LLT	Kessels, R. P. C., Bucks, R. S., Willison, J. R., & Byrne, L. M. T. (2012). *Location Learning Test Herziene Uitgave – Handleiding [LLT Manual]*. Amsterdam: Hogrefe.
Verbal Fluency	Fluency	Luteijn, F., & Barelds, D. P. F. (2004). Groninger Intelligentie Test 2 Handleiding. Amsterdam: Harcourt Assessment B.V.<br>Schmand, B., Houx, P., & De Koning, I. (2012). *Normen voor Psychologische Tests voor gebruik in de Klinische Neuropsychologie.* Sectie Neuropsychologie, Nederlands Instituut voor Psychologen (NIP).
Behavioural Assessment of the Dysexecutive Syndrome	BADS	Wilson, B. A., Alderman, N., Burgess, P.W., Emslie, H., & Evans, J. J. (1996). *BADS: Behavioural Assessment of the Dysexecutive Syndrome.* London: Pearson.<br>Krabbendam, L., & Kalff, A. C. (1997). *BADS-NL. Handleiding [BADS-NL Manual]*. Lisse: Swets & Zeitlinger.

Behavior Rating Inventory of Executive Function, Adult	BRIEF-A	Gioia, G., Isquith, P. K., Guy, S. C., & Kenworthy, L. (2004). *Behavior Rating Inventory of Executive Function (BRIEF): Professional manual.* Lutz, FL: Psychological Assessment Resources. [Dutch translation: Scholte, E., & Noens, I. (2011). *Vragenlijst over executieve functies bij volwassenen (BRIEF-A). Handleiding*]. Amsterdam: Hogrefe.
Dysexecutive Questionnaire	DEX	Krabbendam, L., & Kalff, A. C. (1997). BADS Behavioural Assessment of the Dysexecutive Syndrome Handleiding. Lisse: Swets & Zeitlinger.
d2 Test of Attention	d2	Brickenkamp, R., & Oosterveld, P. (2014). *d2 Aandachts- en concentratietest. Handleiding [Test of Attention. Manual*]. Amsterdam: Hogrefe.
Stroop Color and Word Test	Stroop	Bouma, A., Mulder, J., Lindeboom, J., & Schmand, B. (2012). *Handboek Neuropsychologische Diagnostiek (tweede herziene druk).* Amsterdam: Pearson Assessment and Information B.V.<br>Schmand, B., Houx, P., & De Koning, I. (2012). *Normen voor Psychologische Tests voor gebruik in de Klinische Neuropsychologie.* Sectie Neuropsychologie, Nederlands Instituut voor Psychologen (NIP).
Trail Making Test	TMT	Lezak, M. D., Howieson, D. B., Bigler, E. D., & Tranel. D. (2012). *Neuropsychological Assessment (5th ed.).* New York: Oxford University Press.<br>Schmand, B., Houx, P., & De Koning, I. (2012). *Normen voor Psychologische Tests voor gebruik in de Klinische Neuropsychologie.* Sectie Neuropsychologie, Nederlands Instituut voor Psychologen (NIP).
Emotion Recognition Task	ERT	Kessels, R. P. C., Montagne, B., Hendriks, A. W., Perrett, D. I., & De Haan, E. H. F. (2014). Assessment of perception of morphed facial expressions using the Emotion Recognition Task (ERT): Normative data from healthy participants aged 8-75. *Journal of Neuropsychology,8*(1), 75-93. https://doi.org/10.1111/jnp.12009
Theory-of-Mind test Revised	ToM-test-R	Steerneman, P., & Meesters, C. (2009), *ToM test-R: Handleiding.* Antwerpen/Apeldoorn: Garant.
Toronto Alexithymia Scale-20	TAS-20	Kooiman, C. G., Spinhoven, P., & Trijsburg, R. W. (2002). The assessment of alexithymia: A critical review of the literature and a psychometric study of the Toronto Alexithymia Scale-20. *Journal of Psychosomatic Research, 53*(6), 1083-1090. https://doi.org/10.1016/S0022-3999(02)00348-3
Coping Inventory for Stressful Situations	CISS	Endler, N. S., & Parker, J. D. A. (1999). *Coping Inventory for Stressful Situations (CISS*): Manual. MHS: Toronto. [Dutch translation: De Ridder, D. T. D., & Van Heck, G. L. (2004). *CISS-NL*. Handleiding]. Amsterdam: Pearson.
Questionnaire for the survey of emotion regulation in adults	FEEL-E	Grob, A., & Horowitz, D. (2014). *FEEL-E. Fragebogen zur Erhebung der Emotionsregulation bei Erwachsenen.* Bern: Verlag Hans Huber. [Dutch translation: Punt, D. J. (2014). *Vragenlijst over emotieregulatie bij volwassenen. FEEL-E.*] Amsterdam: Hogrefe.
Adult Behavior Checklist	ABCL	Achenbach, T. M. & Rescorla, L. A. (2003). *Manual for the ASEBA Adult Forms & Profiles*. Burlington, VT: University of Vermont, Research Center for Children, Youth, & Families.
Checklist Individual Strength-20-Revised	CIS-20-R	Vercoulen, J. H. M. M., Alberts, M., & Bleijenberg, G. (1999). Kort instrumenteel: de Checklist Individual Strength (CIS). *Gedragstherapie, 32*(2), 131-136.<br>Vercoulen, J. H. M. M. , Swanink, C. M. A, Fennis, J. F. M., Galama, J. M. D., van der Meer, J. W. M., & Bleijenberg, G. (1994). Dimensional assessment of chronic fatigue syndrome. *Journal of Pychosomatic Research, 38*(5), 383-392. https://doi.org/10.1016/0022-3999(94)90099-X
Fatigue Severity Scale	FSS	Krupp, L. B., LaRocca, N. G., Muir-Nash, J., & Steinberg A. D. (1989). The Fatigue Severity Scale: application to patients with multiple sclerosis and systemic lupus erythematosus. *Archives of Neurology,*

		46(10), 1121-1123.<br>Dutch translation: Rietberg, M. B., van Wegen, E. E. H., & Kwakkel, G. (2010). Measuring fatigue in patients with multiple sclerosis: reproducibility, responsiveness and concurrent validity of three Dutch self-report questionnaires. *Disability and rehabilitation, 32*(22), 1870-1876. https://doi.org/10.3109/09638281003734458<br>Retrieved from: https://meetinstrumentenzorg.nl/instrumenten/fatigue-severity-scale/ [2022, November 14].
Minnesota Multiphasic Personality Inventory-2 Restructured Form	MMPI-2-RF	Ben-Porath, Y. S., & Tellegen, A. (2008). *MMPI-2-RF manual for administration, scoring, and interpretation.* Minneapolis, MN: University of Minnesota Press.<br>Van Der Heijden, P., Derksen, J., Egger, J., Rossi, G., Laheij, M., & Bögels, T. (2013). *MMPI-2-RF. Minnesota Multiphasic Personality Inventory-2 Restructured Form. Handleiding voor Afname, scoring en interpretatie [Manual for administration, scoring and interpretation: Dutch-Flemish translation].* Nijmegen: PEN Tests Publisher.
Beck Depression Inventory-II	BDI-II-NL	Beck, A. T., Steer, R. A., & Brown, G. K. (1996). *Manual for the Beck depression inventory-II*. San Antonio, TX: Psychological Corporation. Van Der Does, A. J. W. (2002). *BDI-II Dutch translation. Handleiding [BDI-II-NL Manual].* The Netherlands, Pearson.<br>Roelofs, J., van Breukelen, G., de Graaf, L. E., Beck, A. T., Arntz, A., & Huibers, M. J. H. (2013). Norms for the Deck Depression Inventory (BDI-II) in a large Dutch community sample. *Journal of Psychopathology and Behavioral Assessment*, *35*(1), 93–98. https://doi.org/10.1007/s10862-012-9309-2
Symptom Checklist-90 Revised	SCL-90-R	Arrindell, W. A., & Ettema, J. H. M. (1986; 2003). *Handleiding bij een multidimensionele psychopathologie-indicator [SCL-90-R. Manual of a multi-dimensional psychopathology indicator].* Amsterdam: Pearson.
